# Association between polycystic ovary syndrome and pregnancy outcomes in GDM: A secondary analysis of the DiGest trial

**DOI:** 10.1210/clinem/dgag026

**Published:** 2026-01-28

**Authors:** Laura C Kusinski, Zhaohui Liu, Sarah Dib, Rebecca Rogers, Amy E Morrison, Danielle L Jones, Claire L Meek

**Affiliations:** Leicester Diabetes Centre and Leicester NIHR Biomedical Research Centre, University of Leicester, Leicester General Hospital, Leicester LE5 4PW, UK; Institute of Metabolic Science—Medical Research Laboratories, University of Cambridge, Cambridge CB2 0QQ, UK; Leicester Diabetes Centre and Leicester NIHR Biomedical Research Centre, University of Leicester, Leicester General Hospital, Leicester LE5 4PW, UK; Leicester Diabetes Centre and Leicester NIHR Biomedical Research Centre, University of Leicester, Leicester General Hospital, Leicester LE5 4PW, UK; Department of Diabetes and Endocrinology, Norfolk and Norwich University Hospitals NHS Trust, Norfolk NR4 7UY, UK; University Hospitals of Leicester NHS Trust, Leicester General Hospital, Leicester LE5 4PW, UK; Institute of Metabolic Science—Medical Research Laboratories, University of Cambridge, Cambridge CB2 0QQ, UK; Leicester Diabetes Centre and Leicester NIHR Biomedical Research Centre, University of Leicester, Leicester General Hospital, Leicester LE5 4PW, UK; Institute of Metabolic Science—Medical Research Laboratories, University of Cambridge, Cambridge CB2 0QQ, UK; University Hospitals of Leicester NHS Trust, Leicester General Hospital, Leicester LE5 4PW, UK; Wolfson Department of Diabetes and Endocrinology, Cambridge Universities NHS Foundation Trust, Cambridge CB2 0QQ, UK

**Keywords:** polycystic ovary syndrome, gestational diabetes mellitus, dietary intervention, neonatal jaundice, insulin use, pregnancy

## Abstract

**Context:**

Polycystic ovary syndrome (PCOS) is a risk factor for gestational diabetes mellitus (GDM) due to shared pathophysiological associations with insulin resistance and adiposity, and may increase the risk of suboptimal perinatal outcomes in GDM. It is unclear if this excess risk upon outcomes is attributable to PCOS, more severe hyperglycemia, higher maternal BMI or reduced efficacy of dietary interventions.

**Objective:**

To assess associations of PCOS, dietary intervention and gestational weight loss with maternal and neonatal outcomes in women with GDM.

**Methods/Design:**

A secondary analysis from the DiGest double-blind randomized controlled trial. Data on self-reported PCOS status were collected at baseline.

**Participants:**

Pregnant women with GDM and BMI ≥25 kg/m^2^ from 8 UK centers (*N* = 425; 50 with PCOS and 375 without PCOS).

**Intervention:**

Reduced-energy intervention diet (1200 kcal/day) or standard-energy control diet (2000 kcal/day) from enrollment (29 weeks) until delivery.

**Main Outcome Measures:**

Pregnancy outcomes, including maternal weight and continuous glucose metrics, physical and dietary data, and infant birthweight and jaundice, were compared between women with and without PCOS using univariate tests and multivariable regression models were applied for adjusted analysis.

**Results:**

Women with GDM and PCOS had similar baseline characteristics, glycemia, BMI, and pregnancy outcomes compared with women with GDM alone, but their infants had higher rates of neonatal jaundice (24.4% vs 8.9%, *P* = .002). Outcomes across the dietary interventions were similar in women with and without PCOS.

**Conclusion:**

In women with GDM, PCOS was not associated with increased risks for most suboptimal pregnancy outcomes or reduced efficacy of a dietary intervention in this cohort where BMI and glycemia were comparable.

Polycystic ovary syndrome (PCOS) is a common endocrine and metabolic disorder affecting 5-18% of women worldwide (1). While different pathophysiological phenotypes of PCOS have increasingly been recognized (2), insulin resistance is a key feature of the condition, affecting 70-75% of women, leading to metabolic syndrome and increasing future risks of type 2 diabetes mellitus (T2DM) (1, 3, 4). Treatment goals in PCOS include achieving and maintaining a healthy weight and intervening to prevent metabolic complications such as T2DM, hypertension, dyslipidemia, and cardiovascular disease.

PCOS in pregnancy is associated with an increased risk of perinatal complications, including gestational hypertension, pre-eclampsia, and preterm birth (1, 3–5). As the state of pregnancy induces insulin resistance, and may worsen pre-existing insulin resistance, women with PCOS may be considered a high-risk group for developing gestational diabetes mellitus (GDM) during their pregnancy (5–9). Although GDM and PCOS commonly co-occur in pregnant women, it is unclear if these two conditions have separate, additive, adverse effects upon pregnancy outcomes.

Several studies have assessed if the presence of PCOS is associated with significantly worse outcomes in women with GDM. However, the literature to date reflects inconsistent and conflicting data, often with very small sample sizes (10). A systemic review by Slouha et al, included some studies showing higher morbidity related to neonatal outcomes for women with coexistent PCOS and GDM, while other studies showed no difference (9, 11, 12). While any increase in risk associated with PCOS might be related to the condition itself, or augmented insulin resistance, any differences could also be explained by other confounding factors. Most previous studies have not adjusted for differences in fertility treatment, maternal age, BMI, dietary choices, the efficacy of dietary management, or baseline glycemia when comparing women with GDM alone to those with coexistant PCOS. One previous study reported that having PCOS does not affect the risks of GDM after adjusting covariates for BMI and age (9). In view of the paucity of data about the risks of coexistent PCOS in GDM, and the efficacy of dietary management, we aimed to investigate how PCOS affects pregnancy outcomes among women with GDM and to determine whether dietary interventions are equally effective in these metabolically complex pregnancies.

## Materials and Methods

### Trial overview

This secondary analysis utilized data from the Dietary Intervention in Gestational Diabetes (DiGest) trial, a multicenter, double-blind randomized controlled trial (RCT) conducted across 8 clinical centers in East Anglia, UK. The primary outcomes of the main DiGest trial were maternal weight change from enrollment to 36 weeks’ gestation and neonatal standardized birthweight. The protocol and original trial findings which evaluated the effects of a reduced-energy diet (1200 kcal/day) vs a standard-energy diet (2000 kcal/day) on maternal and neonatal outcomes in women diagnosed with GDM, have been previously published (13, 14).

The study adhered to the principles of the Declaration of Helsinki and received ethical approval from the National Research Ethics Committee, United Kingdom (Ref: 18/WM/0191) and the NHS Health Research Authority (IRAS 242924; ISRCTN 65152174). All participants provided informed, written consent prior to enrollment in the DiGest trial.

### Study population

Pregnant women aged ≥18 years with an ultrasound-confirmed singleton pregnancy, a GDM diagnosis before 30 + 6 weeks of gestation, and a prepregnancy BMI ≥25 kg/m^2^ were included. GDM was diagnosed using OGTT based on NICE Criteria (15), interim COVID-19 criteria issued by the Royal College of Obstetricians and Gynaecologists (RCOG) (16), or self-monitored blood glucose in those with GDM history (15, 17).

### Trial study timeline

**Figure 1:**
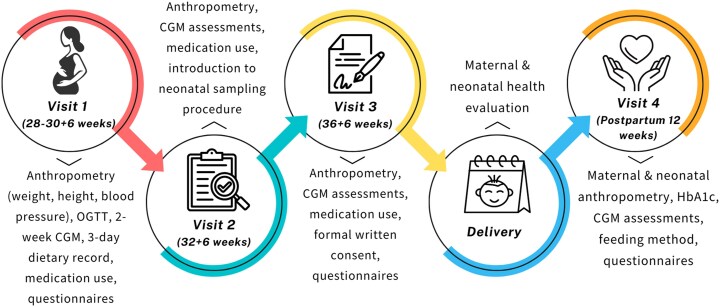
Flowchart of study timeline and key assessments. Abbreviations: CGM, continuous glucose monitoring; OGTT, oral glucose tolerance test.

Eligible participants underwent baseline assessment at 28-30 + 6 weeks and were randomized for dietary intervention from 30 to 32 weeks until delivery. The study included 4 research visits, which are detailed in Fig. 1.

### Randomization, blinding, and dietary intervention characteristics

425 participants were included in the trial and randomized in a 1:1 ratio to either the standard-energy or the reduced-energy diet. A strict double-blind design was used to ensure that participants, clinical teams and researchers were unaware of group allocation. Participants received all preprepared meal boxes providing 2000 or 1200 kcal/day weekly. Meals adhered to pregnancy nutrition and food safety suggestions, ensuring a balanced composition of 40% carbohydrates, 25% protein, and 35% fat while excluding added sugars and artificial additives (18–20).

### PCOS classification

In this study, history of PCOS was self-reported. The Rotterdam Criteria, an international expert consensus first proposed in 2003, are used to define a diagnosis of PCOS in the UK, through the presence of at least 2 of the following 3 features; (1) Oligo-amenorrhea, (2) Clinical or biochemical hyperandrogenism, and (3) polycystic ovarian morphology on ultrasound (21, 22).

### Study outcomes

This secondary analysis compared maternal characteristics at baseline and subsequent maternal and neonatal outcomes between participants with PCOS and those without PCOS, to evaluate the association between PCOS and pregnancy-related parameters. Furthermore, we examined associations of randomized dietary intervention and gestational weight loss with maternal and neonatal outcomes, with PCOS status included as a covariate in regression models. Finally, we examined antenatal dietary intake and physical activity between those with PCOS and those without.

### Maternal outcomes

Maternal outcomes assessed included weight change, BMI, glycemic control (HbA1c and CGM glucose metrics), blood pressure, medication treatments, birth modality, and complications. Gestational weight loss was defined as any decrease in maternal body weight between enrollment and 36 weeks’ gestation. Postpartum glycemia was evaluated by CGM results and HbA1c level. CGM metrics were measured with a masked Dexcom G6 CGM at each study visit. Dietary intake and physical activity were measured through a validated, self-administered online dietary recall software (Intake24; version 2 (2022)) and a validated online recent physical activity questionnaire (RPAQ; Version 08/11/2019) respectively. Participants were asked to complete each questionnaire at baseline (Visit 1; before the dietary intervention began) and again 12-weeks postpartum (Visit 4). RPAQ assesses habitual physical activity over the previous 4 weeks. Intake-24 is a 24-hour dietary recall which we asked participants to complete for 1 weekday and 1 weekend at both time points.

### Neonatal outcomes

Neonatal outcomes assessed included birth weight, birthweight z-score, and classification of small for gestational age (SGA) and large for gestational age (LGA) based on INTERGROWTH-21st centile standards (23, 24). Additional neonatal health indicators included Apgar scores, NICU admission, neonatal jaundice, neonatal hypoglycemia (defined as capillary glucose <2.6 mmol/L, measured at least 30 minutes after birth and within the first 48 hours of life) (25), preterm delivery, congenital anomalies, birth injury, stillbirth, and neonatal death. Cord blood C-peptide levels were measured as a measure of fetal insulin secretion (26).

### Statistical analysis

Maternal characteristics and neonatal outcomes are presented as mean (SD), median [IQR] and *N* (%) where appropriate. Between-group differences by PCOS status were compared using independent *t*-tests, Mann–Whitney *U* tests or χ^2^ tests where appropriate. Multivariable linear or logistic regression models were fitted according to outcome type to examine associations of PCOS status and randomized dietary intervention group with maternal and neonatal outcomes. In additional analysis, gestational weight loss was examined in the same multivariable regression framework to assess its association with maternal and neonatal outcomes in this cohort. Models were adjusted for study center, with additional adjustment for baseline maternal weight when analyzing maternal weight change. PCOS status and in vitro fertilization (IVF) were included as covariates in subsequent models. Linear regression models and logistic regression models were used to assess dietary intervention as well as dietary intake and physical activity. Analysis was performed in SPSS (version 28.0, IBM Corp.) and STATA (v.17.0 StataCorp), with results being considered as statistically significant when *P* < .05.

## Results

### Baseline maternal characteristics

All 425 participants in the DiGest trial had available data on self-reported PCOS status with their baseline characteristics detailed in Table 1. There was no difference in maternal age, BMI, blood pressure, ethnicity, previous GDM between the women with and without PCOS. However, the proportion of participants who conceived through IVF or assisted conception was significantly higher in the PCOS group (16.00% vs 4.55% non-PCOS; *P* = .001).

**Table 1:** Baseline maternal characteristics at enrollment (28-30 weeks)

Variable	*n*	All participants (*n* = 425)	*n*	Non-PCOS (*n* = 375)	*n*	PCOS (*n* = 50)	*P* value
Maternal characteristics							
Age at recruitment, year	425	33.03 ± 5.04	375	33.14 ± 5.05	50	32.25 ± 4.97	.27
Gestational age at diagnosis of GDM, week	414	22.85 ± 6.40	365	22.97 ± 6.45	49	21.99 ± 6.01	.31
Maternal BMI at Visit 1, kg/m^2^	425	35.48 ± 6.31	339	35.38 ± 6.35	46	36.26 ± 5.96	.38
Systolic blood pressure at Visit 1, mmHg	418	115.69 ± 12.47	368	115.74 ± 12.57	50	115.36 ± 11.91	.84
Diastolic blood pressure at Visit 1, mmHg	418	69.29 ± 10.12	368	69.43 ± 10.35	50	68.26 ± 8.19	.44
Ethnicity	425		375		50		.15
White		332 (78.12)		295 (78.67)		37 (74.00)	
Asian		73 (17.18)		60 (16.00)		13 (26.00)	
Black		17 (4.00)		17 (4.53)		0	
Other/Mixed		3 (0.71)		3 (0.80)		0	
Maternal education (<degree)	425	224 (52.71)	375	200 (53.33)	50	24 (48.00)	.48
Index of multiple deprivation decile	412	6.53 ± 2.47	362	6.52 ± 2.43	50	6.60 ± 2.76	.83
Smoking or vaping	422	44 (10.43)	372	38 (10.22)	50	6 (12.00)	.70
Primiparity	385	136 (35.32)	339	118 (34.81)	46	18 (39.13)	.57
In vitro fertilization or assisted conception	424	25 (5.90)	374	17 (4.55)	50	8 (16.00)	.**001**
Gestational diabetes in previous pregnancy	424	122 (28.77)	374	105 (28.07)	50	17 (34.00)	.39
Dietary intervention	425		375		50		.25
Allocation to reduced-energy intervention		214 (0.50)		185 (0.49)		29 (0.58)	
Allocation to standard-energy diet		211 (0.50)		190 (0.51)		21 (0.42)	
GDM diagnosis at enrollment							
Diagnostic methods	425		375		50		.051
OGTT using NICE criteria		217 (51.06)		199 (53.07)		18 (36.00)	
Interim COVID-19 criteria		98 (23.06)		85 (22.67)		13 (26.00)	
Self-monitoring blood glucose devices		110 (25.88)		91 (24.27)		19 (38.00)	
HbA1c, mmol/mol	147	39.00 ± 4.63	130	38.88 ± 4.71	17	39.88 ± 3.95	.41
HbA1c, %	147	5.72 ± 0.42	130	5.71 ± 0.43	17	5.80 ± 0.36	.41
Medication use at 29 weeks							
Metformin	425	94 (22.12)	375	84 (22.40)	50	10 (20.00)	.70
Short-acting insulin	425	38 (8.94)	375	35 (9.33)	50	3 (6.00)	.44
Long-acting insulin	425	101 (23.76)	375	85 (22.67)	50	16 (32.00)	.15
Blood glucose at 29 weeks							
Days of CGM use	361	5.79 ± 2.24	318	5.85 ± 2.21	43	5.35 ± 2.39	.17
Mean of CGM glucose, mmol/L	361	5.77 ± 0.77	318	5.76 ± 0.79	43	5.83 ± 0.66	.61
Mean of CGM glucose, mg/dL	361	103.95 ± 13.89	318	103.81 ± 14.15	43	104.97 ± 11.91	.61
TIR (3.5-7.8 mmol/L) %	361	94.46 [8.56]	318	94.26 [8.79]	43	96.18 [7.21]	.15
TAR (3.5-7.8 mmol/L) %	361	3.20 [7.75]	318	3.21 [8.06]	43	3.20 [5.32]	.59
TBR (3.5-7.8 mmol/L) %	361	0.53 [1.81]	318	0.58 [2.09]	43	0.23 [1.04]	.**019**
Standard deviation	361	1.01 [0.32]	318	1.01 [0.31]	43	0.98 [0.37]	.45
Coefficient of variation	361	17.84 [5.29]	318	17.92 [5.20]	43	17.08 [6.15]	.20

Categorical data are presented as *n* (%), and continuous data as mean ± SD or median [IQR], depending on data distribution. For continuous data, normally distributed variables were analyzed using the independent *t*-test, while non-normally distributed variables were assessed via the Mann–Whitney *U* test. Categorical data were evaluated using the χ^2^ test. Gestational age at diagnosis: the gestational week at which GDM was diagnosed.

Abbreviations: BMI, body mass index; BMR, basal metabolic rate; CGM, continuous glucose monitoring; GDM, gestational diabetes mellitus; OGTT, oral glucose tolerance test; RCOG, Royal College of Obstetricians and Gynaecologists; TIR, time in range; TAR, time above range; TBR, time below range.

Baseline medication and continuous glucose metrics were similar between PCOS and non-PCOS groups, except for time below range. Women with PCOS had a significantly lower time below range compared with those without PCOS (0.23% vs 0.58%, *P* = .019). The absolute difference was minimal: ∼5 minutes per day.

### Association of PCOS with pregnancy outcomes in patients with GDM

Maternal outcomes were analyzed at Visit 3 (36 weeks gestation), delivery and Visit 4 (12 weeks postpartum) (Table 2). There were no differences in maternal anthropometry, CGM metrics, medication at 36 weeks and HbA1c at either time point between the groups. Mean BMI was slightly higher in women with PCOS both at 36 weeks (36.7 ± 5.8 vs 35.6 ± 6.4 kg/m^2^, *P* = .26) and at 12 weeks postpartum (34.4 ± 5.6 vs 32.5 ± 6.2 kg/m^2^, *P* = .09), but these differences did not reach statistical significance. Rates of pre-eclampsia and gestational hypertension were also comparable between women with and without PCOS (both *P* > .05).

**Table 2:** Maternal outcomes at 36 weeks of pregnancy, delivery, and 3 months postpartum in participants with and without PCOS

Variable	*n*	Non-PCOS (*n* = 375)	*n*	PCOS (*n* = 50)	*P* value
Maternal anthropometry at 36 weeks					
Maternal weight, kg	342	95.56 ± 20.02	46	97.72 ± 16.48	.23
Maternal BMI, kg/m^2^	319	35.58 ± 6.38	44	36.72 ± 5.78	.26
Maternal weight change 29-36 weeks, kg	342	0.60 [3.90]	46	1.00 [5.10]	.34
Systolic blood pressure, mmHg	282	117.84 ± 13.11	38	120.21 ± 13.66	.30
Diastolic blood pressure, mmHg	282	71.49 ± 10.39	38	73.11 ± 9.29	.36
Blood glucose at 36 weeks					
Days of CGM use	201	5.48 ± 2.19	26	5.58 ± 2.21	.83
Mean of CGM glucose, mmol/L	201	5.81 ± 0.81	26	5.71 ± 0.68	.54
TIR (3.5-7.8 mmol/L) %	201	92.85 [10.83]	26	94.57 [5.67]	.59
TAR (3.5-7.8 mmol/L) %	201	4.77 [10.17]	26	3.68 [3.50]	.75
TBR (3.5-7.8 mmol/L) %	201	0.46 [1.66]	26	0.21 [1.28]	.44
Standard deviation	201	1.03 [0.45]	26	0.99 [0.24]	.62
Coefficient of variation	201	17.94 [5.33]	26	17.20 [3.34]	.77
Medication use at 36 weeks					
Metformin use	270	79 (29.26)	40	9 (22.50)	.38
Short-acting insulin use	270	35 (12.96)	40	6 (15.00)	.72
Long-acting insulin use	271	87 (32.10)	40	17 (42.50)	.19
Maternal outcomes at delivery					
Gestational age at birth, week	338	38.43 ± 1.32	46	38.48 ± 1.06	.80
Cesarean section	375	159 (42.40)	50	23 (46.00)	.63
Maternal outcomes before or at delivery					
Pre-eclampsia	338	7 (2.07)	45	0	.33
Gestational hypertension	338	16 (4.73)	45	3 (6.67)	.58
Preterm delivery	338	32 (9.47)	46	4 (8.70)	.87
Maternal weight and BMI at 3 months postpartum					
Maternal weight, kg	229	87.03 ± 18.68	32	91.66 ± 16.53	.19
Maternal BMI, kg/m^2^	229	32.47 ± 6.23	32	34.35 ± 5.61	.09
Blood glucose at 3 months postpartum					
HbA1c, mmol/mol	219	36.67 ± 4.01	30	37.57 ± 3.88	.24
HbA1c, %	219	5.51 ± 0.37	30	5.59 ± 0.35	.24
Days of CGM use, days	375	2.56 ± 3.16	50	1.91 ± 2.94	.17
Mean of CGM glucose, mmol/L	176	6.27 ± 0.70	21	6.33 ± 1.07	.73
TIR (3.5-7.8 mmol/L) %	176	90.32 [10.94]	21	91.49 [13.97]	.49
TAR (3.5-7.8 mmol/L) %	176	9.07 [12.02]	21	8.34 [15.81]	.52
TBR (3.5-7.8 mmol/L) %	176	0.05 [0.41]	21	0.00 [0.67]	.79
Standard deviation	176	1.03 [0.26]	21	0.98 [0.27]	.71
Coefficient of variation	176	16.40 [3.72]	21	16.93 [5.92]	.86

Categorical data are presented as *n* (%), and continuous data as mean ± SD or median [IQR], depending on data distribution. For continuous data, normally distributed variables were analyzed using the independent *t*-test, while non-normally distributed variables were assessed via the Mann–Whitney *U* test. Categorical data were evaluated using the χ^2^ test.

Neonatal outcomes evaluated at delivery and 12-weeks postpartum are summarized in Table 3. No significant differences were found in any of the outcomes examined apart from neonatal jaundice which occurred more frequently in the PCOS group (24.44% vs 8.90%, *P* = .002).

**Table 3:** Neonatal outcomes in participants with and without PCOS

Variable	*n*	Non-PCOS (*n* = 375)	*n*	PCOS (*n* = 50)	*P* value
Perinatal outcomes					
Birthweight, g	337	3282.92 ± 485.65	46	3284.83 ± 403.20	.98
Birthweight z-score (intergrowth)	336	0.45 ± 0.99	46	0.42 ± 0.85	.86
Birthweight centile (intergrowth)	336	66.57 [42.85]	46	67.47 [34.48]	.74
LGA (intergrowth)	336	68 (20.24)	46	6 (13.04)	.25
SGA (intergrowth)	336	16 (4.76)	46	1 (2.17)	.43
Apgar score <7 at 1 minute	336	22 (6.55)	46	4 (8.70)	.59
Apgar score <7 at 5 minutes	336	6 (1.79)	46	1 (2.17)	.85
Cord blood c-peptide, pmol/L	82	228.00 [235.00]	16	162.00 [173.00]	.19
Neonatal hypoglycemia	337	16 (4.75)	45	3 (6.67)	.58
Neonatal jaundice	337	30 (8.90)	45	11 (24.44)	.**002**
NICU admission	337	37 (10.98)	45	3 (6.67)	.38
Neonatal outcomes at 3 months postnatal					
Baby weight, g	228	6022.28 ± 898.02	28	5997.89 ± 718.20	.89
Infant receiving breast milk	242	143 (59.09)	33	20 (60.61)	.87
Infant receiving only formula milk	241	99 (41.08)	33	13 (39.39)	.85
Infant receiving only breast milk	242	92 (38.02)	33	13 (39.39)	.88

Categorical data are presented as *n* (%), and continuous data as mean ± SD or median [IQR], depending on data distribution.

Abbreviations: LGA, large for gestational age; SGA, small for gestational age.

### Adjusted regression analysis of dietary intervention on maternal and neonatal outcomes

Table 4 presents the primary endpoints of the trial, which were maternal weight change and neonatal standardized birthweight, along with selected neonatal outcomes prespecified in the trial protocol. Long-acting insulin requirement, the only secondary outcome showing a significant between-group difference in the original trial, was also analyzed for potential modification by PCOS status.

**Table 4:** Adjusted effects of dietary intervention on maternal and neonatal outcomes in multivariable models

Variables	Model 1		Model 2		Model 3	
	Intervention effect (95% CI)	*P* value	Intervention effect (95% CI)	*P* value	Intervention effect (95% CI)	*P* value
Maternal outcomes						
Weight change (Visit 3—Visit 1)	*B*; −0.20 (−1.01, 0.61)	.63	*B*; −0.20 (−1.01, 0.61)	.63	*B*; −0.19 (−1.00, 0.63)	.65
Long-acting insulin use at Visit 3	OR; 0.60 (0.37, 0.99)	.**044**	OR; 0.59 (0.36, 0.97)	.**038**	OR; 0.57 (0.34, 0.94)	.**028**
Neonatal outcomes						
Birthweight z-score (intergrowth)	*B*; 0.01 (−0.19, 0.20)	.96	*B*; 0.01 (−0.19, 0.20)	.96	*B*; 0.00 (−0.19, 0.20)	.97
Birthweight centile (intergrowth)	*B*; −0.70 (−6.13, 4.73)	.80	*B*; −0.70 (−6.14, 4.75)	.80	*B*; −0.70 (−6.17, 4.76)	.80
LGA (intergrowth)	OR; 1.11 (0.66, 1.86)	.70	OR; 1.12 (0.67, 1.89)	.67	OR; 1.11 (0.66, 1.87)	.69
Cord blood C-peptide	*B*; −61.68 (142.38, 19.02)	.13	*B*; −58.29 (−140.56, 23.99)	.16	*B*; −62.44 (145.49, 20.61)	.14
NICU admission	OR; 1.36 (0.69, 2.68)	.38	OR; 1.38 (0.70, 2.73)	.35	OR; 1.38 (0.70, 2.72)	.36

Values reaching the threshold of significance are highlighted in bold. Linear regression was used for continuous outcomes, and logistic regression for binary outcomes. Results are presented as regression coefficients (*B*) for continuous outcomes and odds ratios (OR) for binary outcomes, with 95% confidence intervals. Model 1 was adjusted for, study centers, and for baseline maternal weight when analyzing the outcome of maternal weight change. Model 2 included the same adjustments as Model 1, with the addition of PCOS status. Model 3 included all covariates in Model 2, with additional adjustment for IVF.

As shown in Table 4, participants receiving the low-energy dietary intervention did not show significant differences in maternal weight change after adjustment for study center, baseline maternal weight at Visit 1, PCOS and IVF. In Model 1, which adjusted for study center, the low-energy diet was significantly associated with a reduced likelihood of long-acting insulin use at Visit 3 (OR 0.60, 95% CI 0.37-0.99, *P* = .044). This association also remained significant after further adjustment for PCOS in Model 2 (OR 0.59, 95% CI 0.36-0.97, *P* = .038) and for PCOS and IVF in Model 3 (OR 0.57, 95% CI 0.34-0.94, *P* = .028), with consistent effect estimates across all 3 models. In contrast, no significant effects of dietary intervention were observed on any of the neonatal outcomes.

### Adjusted regression analysis of maternal weight loss on maternal and neonatal outcomes

The outcomes assessed in Table 5 were aligned with those analyzed in Table 4 to allow for comparability of findings across models. Of the 389 women with sufficient data to assess weight change between enrollment and 36 weeks gestation, 154 (39.6%) were categorized as having a weight loss. This number remained consistent across all models, with 154 women included in each model after accounting for missing covariate data. This weight loss, defined independently of dietary intervention and treated as an exploratory exposure, was not significantly associated with long-acting insulin use at Visit 3 in any model (Table 5). However, weight loss was significantly associated with a reduced likelihood of LGA (all *P* < .05 across all models). These associations remained significant after adjustment for study center, dietary intervention, PCOS and IVF. No significant associations were found between weight loss and other neonatal outcomes.

**Table 5:** Adjusted effects of weight loss on maternal and neonatal outcomes in multivariable models

Variables	Model 1		Model 2		Model 3	
	Intervention effect (95% CI)	*P* value	Intervention effect (95% CI)	*P* value	Intervention effect (95% CI)	*P* value
Maternal outcomes						
Long-acting insulin use at Visit 3	OR; 0.69 (0.41, 1.16)	.16	OR; 0.69 (0.41, 1.17)	.17	OR; 0.69 (0.41, 1.18)	.17
Neonatal outcomes						
Birthweight z-score (intergrowth)	*B*; −0.16 (−0.37, 0.04)	.11	*B*; −0.16 (−0.37, 0.04)	.12	*B*; −0.16 (−0.37, 0.04)	.12
Birthweight centile (intergrowth)	*B*; −4.49 (−10.19, 1.22)	0.12	*B*; −4.46 (−10.17, 1.26)	.13	*B*; −4.47 (−10.20, 1.27)	.13
LGA (intergrowth)	OR; 0.52 (0.29, 0.93)	.**027**	OR; 0.51 (0.29, 0.92)	.**025**	OR; 0.51 (0.28, 0.91)	.**024**
SGA (intergrowth)	OR; 0.43 (0.13, 1.43)	.17	OR; 0.43 (0.13, 1.41)	.16	OR; 0.40 (0.12, 1.36)	.14
Cord blood C-peptide	*B*; −39.56 (−121.60, 42.48)	.34	*B*; −40.32 (−122.78, 42.14)	.33	*B*; −41.02 (−124.15, 42.10)	.33
NICU admission	OR; 0.81 (0.38, 1.73)	.58	OR; 0.80 (0.38, 1.72)	.57	OR; 0.80 (0.37, 1.71)	.56

Values reaching the threshold of significance are highlighted in bold. Weight loss was defined as any decrease in maternal weight between enrollment and 36 weeks’ gestation. Participants were excluded if they had no weight data at 36 weeks, or if the pregnancy ended before 36 weeks due to stillbirth, neonatal death, or maternal death. Outcomes were analyzed using linear regression for continuous variables and logistic regression for binary variables, with results expressed as regression coefficients (*B*) or odds ratios (OR) and 95% confidence intervals. Model 1 assessed the effect of weight loss, adjusted for study centers and dietary intervention. Model 2 included the same adjustments as Model 1, with the addition of PCOS status. Model 3 included all covariates in Model 2, with additional adjustment for IVF.

### Dietary patterns and physical activity during pregnancy

The number of recalls for dietary assessment and physical activity assessment were too low for detailed analysis. However, there were no obvious differences in maternal diet or physical activity metrics between women with and without PCOS (Tables S1 and S2 (27)).

## Discussion

### Statement of main findings

In this cohort, where women with PCOS were statistically similar to other women with GDM in terms of BMI, and glycemia, PCOS was not associated with increased perinatal risks. The reduced-energy dietary intervention was associated with lower use of long-acting insulin across the cohort, and gestational weight loss was associated with reduced odds of LGA. Both associations changed little after adjustment for PCOS status, but should be interpreted cautiously given the observational nature of this secondary analysis and the modest sample size. However, neonatal jaundice had a higher incidence in women with PCOS, an unexpected finding that should be interpreted cautiously and validated in larger cohorts. Women with PCOS and GDM who received a reduced-energy diet in pregnancy had similar benefit to women with GDM alone, suggesting that dietary management is effective even in women with more metabolically complex pregnancies.

### Relation to previous research

Despite evidence linking PCOS to increased pregnancy complications, data remain limited for women with PCOS who also have GDM and elevated prepregnancy BMI. Most existing studies are retrospective observational analyses or meta-analysis with considerable heterogeneity in study populations (eg, variations in prepregnancy BMI or GDM status) and inconsistent intervention strategies (eg, dietary intervention, physical activities). Few prospective randomized trials have focused specifically on this high-risk subgroup, limiting the ability to draw firm conclusions about pregnancy outcomes.

### Reduced association of PCOS on pregnancy outcomes under GDM management

Studies across the last 2 decades have consistently found an increased risk of hypertensive disorders and pregnancy complications associated with PCOS. Foroozanfard et al evaluated obstetric and neonatal outcomes of 261 GDM women, with and without a diagnosis of PCOS. Women with PCOS and GDM had more than 2-fold increase in risk of pre-eclampsia and pregnancy-induced hypertension, and 3-fold increase in neonatal hypoglycemia (28). Similar results were shown by Aktun et al, with that the risk of pregnancy-induced hypertension increased by 2.4-fold, pre-eclampsia by 2-fold, and neonatal hypoglycemia by 3.2-fold in the group with PCOS and GDM in comparison to GDM alone (12). By contrast, in our trial, the incidence of gestational hypertension and pre-eclampsia was low in both groups and did not differ significantly between women with and without PCOS. Given the small size of the PCOS subgroup and the low event rates, our study had limited power to detect any additional increase in the risk of gestational hypertension or pre-eclampsia associated with PCOS, as suggested in previous studies.

A meta-analysis by Khomami et al (29) reported increased risks of preterm birth, low birth weight, and fetal growth restriction among women with PCOS, but no significant association with LGA or macrosomia. Also, in subgroup analysis limited to pregnancies complicated by GDM, the association between PCOS and preterm birth was no longer statistically significant, consistent with our findings, where there was no significant difference in gestation at birth. Qin et al (30) similarly found that PCOS was associated with higher risks of preterm birth, low birth weight, and NICU admission. Although women with PCOS typically have higher BMI and a greater likelihood of developing GDM, which would theoretically increase the risk of LGA, the authors suggested that placental dysfunction and impaired fetal growth may counteract these metabolic effects (29, 31). In addition, the use of metformin before and during pregnancy in women with PCOS may contribute to the risk of SGA infants and complicate the interpretation of PCOS-related effects on neonatal outcomes (32). These hypotheses imply that the GDM related metabolic environment and clinical management may attenuate PCOS specific risks (29, 31, 32).

Interestingly, Valdimarsdottir et al (9) observed that while PCOS and GDM were each independently associated with adverse outcomes, no additive risk was found when the conditions coexisted, comparable to our study, suggesting that the effects of both GDM and PCOS are mediated through a similar pathophysiological pathway, perhaps characterized by obesity, insulin resistance and hyperglycemia. Fougner et al (33) reported that among women with PCOS, those who developed GDM did not experience higher rates of pregnancy complications. Chen et al (34) also showed in a large study examining over 1 million pregnancies, PCOS increased the risk of preterm birth in women with GDM, but had no significant association with LGA or SGA in women with either GDM or T2DM. However, a recent systematic review by Slouha et al (11) summarized that the coexistence of PCOS and GDM may increase additional maternal and neonatal risk, with higher rates of gestational hypertension and low birth weight compared with GDM alone. These inconsistencies may reflect differences in study design, PCOS diagnosis (35), PCOS phenotypes (2, 3), conception methods, or the timing and intensity of clinical management.

### Increased risk of neonatal jaundice among infants born to women with GDM and PCOS

Our data shows elevated levels of neonatal jaundice in women with PCOS. A similar association was reported by Rees et al (36) based on a UK medical database, with an adjusted odds ratio of 1.20, further supporting a potential link between PCOS and altered neonatal bilirubin metabolism. Notably, their study also mentioned a significantly increased risk of preterm birth in women with PCOS. However, since their analysis did not adjust for prematurity, which is a known risk factor for neonatal jaundice, it remains unclear whether the increased risk of jaundice was independently associated with the exposure or partly confounded by preterm birth. Additionally, the Rees study was conducted in a general obstetric population, whereas our cohort consisted exclusively of women with GDM and elevated prepregnancy BMI, representing a metabolically higher-risk subgroup. These differences limit direct comparability, but they also raise the possibility that the elevated risk of jaundice in this subgroup may reflect the combined metabolic burden of GDM and PCOS. In contrast, Dehghani Firoozabadi et al (37) found no differences in neonatal jaundice across different PCOS phenotypes. However, their study lacked a non-PCOS comparison group, limiting generalizability to broader populations. These observations underscore the need for further investigation into how maternal metabolic phenotypes, including PCOS subtypes and levels of glycemic control, influence neonatal jaundice risk. Clarifying these pathways could support more targeted monitoring and early intervention strategies in high-risk pregnancies.

### Addressing pharmacologic needs in GDM by dietary intervention

The main DiGest study demonstrated that a low-energy dietary intervention significantly reduced the requirement for long-acting insulin among women with GDM (14). Building upon this, our secondary analysis assessed whether this effect remained independent after adjusting for maternal background factors. Although PCOS and IVF were not independently associated with insulin use in our analysis, their sequential inclusion in the regression models strengthened the inverse association between dietary intervention and long-acting insulin use, suggesting that these variables may reflect underlying metabolic differences that influence how women respond to the intervention.

Previous studies have suggested that women with both GDM and PCOS may represent a subgroup with greater pharmacologic needs during pregnancy. Manoharan and Wong (38) reported significantly higher use of both metformin and insulin among women with GDM and comorbid PCOS, with a proportion requiring combination therapy. They also found that PCOS was associated with higher fasting glucose during OGTT, and that BMI was significantly correlated with all glucose parameters in the PCOS group, whereas this correlation was limited to fasting glucose in women without PCOS. These findings suggest that PCOS may exacerbate gestational glycemic dysregulation, particularly in the presence of obesity, highlighting the importance of individualized weight and glycemic monitoring. Similarly, Atkins et al (39) observed that PCOS significantly increased the likelihood of insulin use in pregnant women with T2DM, further supporting its role as a marker of insulin resistance and metabolic burden.

Our results offer an additional perspective on the effectiveness of dietary intervention. Although PCOS and IVF were not independently associated with insulin use in our analysis, sequential adjustment for these variables resulted in only modest shifts in effect estimates. This suggests that while PCOS and IVF may be associated with insulin requirements in pregnancy, their role in the observed association between dietary allocation and long-acting insulin use appeared limited within this dataset.

### Association between gestational weight management and neonatal birthweight

Although maternal weight loss was not associated with most neonatal outcomes, it consistently showed a significant inverse association with LGA across all models. This suggests that moderate weight reduction in late pregnancy in women with GDM and PCOS may reduce the risk of excessive fetal growth, without adversely affecting other neonatal outcomes, including birthweight z-score, SGA, cord blood C-peptide, or NICU admission. These results indicate that moderate maternal weight loss may have a selective rather than broad association with fetal growth. Also, the main DiGest trial supported that late pregnancy weight loss in women with GDM was associated with improved glycemic profiles and lower rates of LGA, without increasing the risk of SGA (14).

A previous study by Jiao et al (40) has shown that neonatal birth weight among non-PCOS women is comparable to that of women with PCOS and a normal prepregnancy BMI, with the elevated risk of macrosomia largely restricted to those in the overweight or obese PCOS subgroup. This underscores the importance of effective weight management in reducing macrosomia risk among women with PCOS and prepregnancy obesity. Additionally, after adjusting for maternal BMI and gestational weight gain, Fougner et al found that the presence of GDM does not appear to significantly affect neonatal birthweight z-scores or macrosomia incidence in PCOS pregnancies, suggesting that maternal weight status and metabolic control are the key determinants of neonatal outcomes in this population (33).

### Implications for clinical practice

PCOS is an established risk factor for GDM. Lim et al (41) reported that while lifestyle interventions, including diet and physical activity, can reduce the overall risk of GDM, their effectiveness in women with PCOS appears limited. Takele et al (42) and Quotah et al (43) found that early combined interventions in metabolically high-risk women can substantially reduce GDM incidence. However, in clinical practice, women with PCOS often exhibit insulin resistance and glucose intolerance before or early in pregnancy, yet these risks are frequently unaddressed until GDM is diagnosed later in gestation (44). Therefore, while primary prevention is important, a substantial proportion of women with PCOS will inevitably develop GDM and require effective, evidence-based management strategies after diagnosis.

Quaresima et al (45) pointed out that, once GDM is diagnosed in women with PCOS, healthy diet and regular physical activity should be promptly recommended, with insulin therapy introduced when glycemic targets are not achieved. Kim et al (46) further highlighted that nutritional education and promotion of physical activity are key components of lifestyle management in women with PCOS and in those with GDM, and may contribute to better metabolic health and lower long-term diabetes risk. The present findings suggest that nutritional management remains effective even when metabolic risk is already present, offering a practical option to reduce insulin use and support maternal and neonatal outcomes. Nonetheless, clearer guidance on the optimal timing and intensity of postdiagnosis interventions in this high-risk group is still lacking. Future research should determine how different maternal metabolic phenotypes modify treatment response and long-term outcomes. Larger studies with detailed metabolic phenotyping are also needed to clarify the risks associated with PCOS in pregnancy and to optimize management strategies.

### Strengths and limitations

This secondary data analysis used high-quality clinical data from the DiGest trial. The trial followed a double-blind design with standardized protocols and consistent data collection across a diverse ethnic and sociodemographic population, which strengthened the reliability of the findings. Also, the dataset encompassed multiple time points during pregnancy and included detailed pregnancy outcomes, providing a strong foundation for this secondary analysis. Importantly, the dataset comprised systematically recorded PCOS status and key confounders, allowing for accurate adjustment in multivariable models and supporting the validity of the dietary intervention effect estimates.

Several limitations should also be considered when interpreting the results. PCOS requires a clinical diagnosis, following the Rotterdam Criteria. We did not have clinical information to fulfill these criteria in the participants in our study, and the diagnosis of PCOS was self-reported. Some people with GDM may have had undiagnosed PCOS. We had markedly unequal cohort sizes, with only 50 women with PCOS compared with 375 without a known PCOS diagnosis. This large imbalance reduces the statistical power of subgroup comparisons, particularly for less common pregnancy outcomes, so nonsignificant differences between groups should not be interpreted as evidence that no true differences exist. Also, the small subgroup sizes precluded adjustment for potential confounders in the comparison between women with and without PCOS. In addition, due to the self-reported nature of the condition in our study we are lacking data relating to method of diagnosis, presenting features and duration of PCOS, so we were unable to characterize PCOS according to phenotype. Although we recorded metformin and insulin use from the time of enrollment, the lack of early pregnancy or preconception medication data limited our ability to fully account for treatment-related confounders. As metformin is frequently prescribed to women with PCOS during the preconception or pregnancy period and may influence fetal growth, this limitation may have led to residual confounding in the observed associations between PCOS and neonatal outcomes (5, 32).

We know there is significant heterogeneity in the phenotypic expressions of PCOS (47). These participants were individuals with self-reported PCOS who had become pregnant. Since PCOS is associated with infertility, women with PCOS may have a selection bias toward milder cases or specific phenotypic types of PCOS with fewer reproductive sequelae. Our findings therefore may be less applicable to women with more marked infertility or more severe phenotypes (1, 48, 49).

Although the association between PCOS and neonatal jaundice was statistically significant, the result should be interpreted with caution due to the small sample size and that jaundice was not a prespecified endpoint of the study. Moreover, as we examined multiple maternal and neonatal outcomes without formal correction for multiple testing, this association may partly reflect chance and should be regarded as exploratory and in need of confirmation in future studies.

The COVID-19 pandemic led to changes in the diagnostic approach for GDM, with some participants diagnosed using interim COVID-19 criteria or self-monitored glucose devices instead of the standard OGTT (50). There was also a borderline significant difference in the diagnostic methods used between women with and without PCOS (*P* = .051), raising the possibility that GDM diagnosis may have varied between subgroups. Logistical challenges occurred in the assessment of biochemical parameters during our study, as a result of the COVID-19 pandemic. Therefore, we have fewer prenatal results available in comparison to our postnatal outcomes, notably for HbA1c results. This leads to challenges in assessing significant differences in the outcomes between our cohorts, and the contribution of baseline glycemia to these outcomes and limits the scope of longitudinal glycemic outcome assessment.

## Conclusions

In this cohort, women with PCOS and GDM did not have worse maternal or neonatal outcomes than women without PCOS, although a higher incidence of neonatal jaundice was noted in the PCOS group and should be interpreted with caution given the small sample size. Across the cohort, dietary intervention was associated with lower use of long-acting insulin, and gestational weight loss was associated with reduced odds of LGA, with no notable differences in other outcomes. These associations remained similar after adjustment for PCOS and IVF, suggesting that these background factors were not major contributors to the observed relationships. PCOS was not associated with increased risks for most suboptimal pregnancy outcomes or reduced efficacy of a dietary intervention in this cohort where BMI and glycemia were comparable.

## Data Availability

Anonymized participant data is only available upon request from the corresponding author and subject to approval from the trial steering committee.

## Clinical trial registry

ISRCTN 65152174; https://www.isrctn.com/ISRCTN65152174.
